# Array comparative genomic hybridisation-based identification of two imbalances of chromosome 1p in a 9-year-old girl with a monosomy 1p36 related phenotype and a family history of learning difficulties: a case report

**DOI:** 10.1186/1752-1947-2-355

**Published:** 2008-11-19

**Authors:** Gregory J Fitzgibbon, Jill Clayton-Smith, Siddharth Banka, Susan J Hamilton, Margaret M Needham, Jonathan K Dore, Jake T Miller, Gareth D Pawson, Lorraine Gaunt

**Affiliations:** 1 Regional Cytogenetics Unit, Saint Mary's Hospital, Hathersage Road, Manchester, M13 0JH, UK; 2 Clinical Genetics Department, Saint Mary's Hospital, Hathersage Road, Manchester, M13 0JH, UK

## Abstract

**Introduction:**

Monosomy 1p36 is one of the most common terminal deletion syndromes, with an approximate incidence of 1 in every 5000 live births. This syndrome is associated with several pronounced clinical features including characteristic facial features, cardiac abnormalities, seizures and mental retardation, all of which are believed to be due to haploinsufficiency of genes within the 1p36 region. The deletion size varies from approximately 1.5 Mb to 10 Mb with the most common breakpoints located at 1p36.13 to 1p36.33. Over 70% of 1p36 deletion patients have a true terminal deletion. A further 7% have interstitial deletions and a proportion have a derivative chromosome 1 where the 1p telomere is replaced by material from another chromosome, either as a result of a de-novo rearrangement or as a consequence of malsegregation of a balanced parental translocation at meiosis.

**Case presentation:**

Array comparative genomic hybridisation analysis of a 9-year-old Caucasian girl presenting with dysmorphic facial features and learning difficulties, for whom previous routine karyotyping had been normal, identified two submicroscopic rearrangements within chromosome 1p. Detection of both an insertional duplication of a region of 1p32.3 into the subtelomeric region of the short arm of a chromosome 1 homologue and a deletion within 1p36.32 of the same chromosome instigated a search for candidate genes within these regions which could be responsible for the clinical phenotype of the patient. Several genes were identified by computer-based annotation, some of which have implications in neurological and physical development.

**Conclusion:**

Array comparative genomic hybridisation is providing a robust method for pinpointing regions of candidate genes associated with clinical phenotypes that extend beyond the resolution of the light microscope. This case report provides an example of how this method of analysis and the subsequent reporting of findings have proven useful in collaborative efforts to elucidate multiple gene functions from a clinical perspective.

## Introduction

Unbalanced chromosome rearrangements account for a significant proportion of phenotypic abnormalities within the population. Varying levels of dysmorphism and mental retardation are attributable to deletions or amplifications of functional regions within the genome. Subtelomeric deletions are a frequent cause of phenotypic abnormalities [1]. The gene rich content of the subtelomeres in comparison to the rest of the human genome [2] makes them an analytical priority for conventional and molecular cytogenetic investigations [3].

Monosomy 1p36 is one of the most common and well characterised subtelomeric deletion syndromes and is associated with mental retardation and multiple congenital abnormalities [4]. The estimated prevalence of this disorder is 1 in 5000 live births [5]. The clinical features of monosomy 1p36 include deep-set eyes, straight eyebrows positioned low on the supra-orbital ridges, asymmetric ears, pointed chin and a flat nasal bridge. Additional features include developmental delay, cardiomyopathy, hearing impairment and seizures (reviewed in [6]), [7]. The 1p36 region is thought to contain numerous tumour-suppressor genes, which is evident by the association of a variety of neoplasms in patients with deletions of this chromosome location [8,9].

Interstitial duplications of the short arm of chromosome 1 are rare with a varied phenotype. A large duplication of chromosome 1 (1p31–1p35) has been previously reported in a baby with low birth weight and post natal growth retardation, congenital heart disease, mid face hypoplasia, ambiguous genitalia and hypoplasia of the phalanges. This child survived for only a short time after birth [10]. A case involving a direct duplication of chromosome 1, dir dup(1)(p21.2–p32) was described in a boy with multiple congenital anomalies. These included microcephaly, malformed ears, anteverted nostrils, convergent squint, micrognathia, hypoplasia of the terminal phalanges, clinodactyly of the fifth fingers, simian creases, left inguinal hernia, cryptorchidism, and severe postnatal growth retardation [11]. A third case involving a duplication of 1p22.3–1p32.3 was described for a child presenting with sex-reversal, mid face abnormalities, an eczema-like skin condition and severe growth retardation [12]. Finally, a 1p31–1p34.1 duplication was reported in a child with craniosynostosis, developmental delay and gut malrotation [13].

Array comparative genomic hybridisation (aCGH) is a high-throughput method used to detect small copy number changes within the genome that are not always visible by conventional microscopy. This is achieved by competitive binding of patient DNA against a 'normal' control DNA to a target sequence which is anchored to a glass slide. The high-dimensional data produced from all of the genomic target sequences is interpreted using an integrated suite of bioinformatic tools.

In comparison to conventional cytogenetic techniques, aCGH enables a more precise examination of the genome, allowing the detection of previously unidentified submicroscopic imbalances and also redefinition of breakpoints in previously identified chromosome imbalances. The ability to accurately map breakpoints means that aCGH has become an efficient strategy in identifying candidate chromosomal loci and genes. This case report highlights the use of aCGH not only to detect submicroscopic deletions and duplications, but also to localise disease gene regions for subsequent candidate gene identification.

## Case presentation

A 9-year-old Caucasian girl presented to the genetic clinic with mild to moderate learning disability. She was noted to have numerous dysmorphic features including a small mouth with heaped up palate, a small chin and a small over folded ear, straight eyebrows, fifth finger clinodactyly and short toes. She had required grommets for treatment of glue ear and had hypermetropia. An 11-week scan detected nuchal oedema and ventriculomegaly was detected on a later antenatal scan. She was of normal birth weight but had feeding difficulties including reflux from birth. A delayed ability to sit, speech delay, and late walking at 22 months were also noted. Height and weight were both in the 50–75^th ^centile at four and a half years old with an occipital-frontal circumference in the 98^th ^centile. A diagnosis of Di George syndrome was initially suspected and she was referred for 22q11 fluorescence in situ hybridisation (FISH) analysis and routine cytogenetic investigations, both of which revealed an apparently normal female karyotype (46, XX). Examination of the subject's mother revealed some mild dysmorphism, clinodactyly of the fifth finger and mild learning disability. The maternal grandmother was also assessed and was phenotypically normal. The proband's father was not available for assessment, however it was reported that he had fathered another child of different maternal origin who also displayed growth and developmental problems.

The proband was the eldest of three sisters, all of whom had different fathers. The second sister had intra uterine growth retardation and low birth weight (4lb 3oz at 37 weeks), with all growth parameters <0.4^th ^centile at 23 months. Similar dysmorphic features as evident in the proband were noted, including small mouth and chin. Cutis marmorata, long slim feet, complete 2/3 syndactyly of the toes and mild to moderate learning disability were also present. Conventional cytogenetic analysis of this sibling revealed a normal female karyotype.

The youngest sister showed an apparently normal development, with mild dysmorphia similar to that of her mother. Currently, the proband's mother is pregnant with a fourth child of different paternity, and has undergone prenatal diagnosis, the conventional cytogenetic analysis for which has revealed a normal female karyotype.

aCGH was performed on DNA from the proband using the Version 2 CytoChip™ from BlueGnome. These arrays are constructed using clones from the widely validated Roswell Park (RP) Cancer Institute Bacterial Artificial Chromosome (BAC) library. The quality control (QC) parameters for diagnostic reporting of this experiment were met with a spot inclusion of 99.71% and a standard deviation (SD) of the autosome value of 0.061 (failure thresholds are <95% and >0.075, respectively) from a 5 μm scan. The scan results, processed by BlueFuse software (BlueGnome), revealed two major genomic imbalances. The first was a five BAC clone deletion between 1.4 and 2 Mb long, mapping to 1p36.32. These clones were (in order of most distal): RP3-395M20, RP11-333E3, RP4-785P20, RP11-46F15 and RP1-286D6.

**Figure 1:**
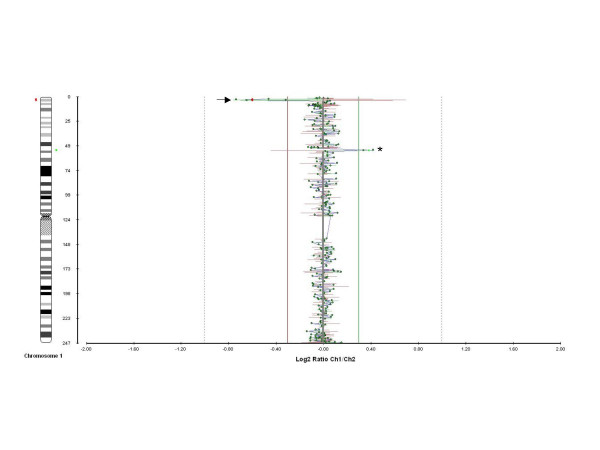
**Array comparative genomic hybridisation scatter plot**. An array comparative genomic hybridisation scatter plot for chromosome 1 generated by BlueFuse microarray analysis software (BlueGnome) showing the five Bacterial Artificial Chromosome clone deletions (BACs RP3-395M20, RP11-333E3, RP4-785P20, RP11-46F15 and RP1-286D) within 1p36.32 (arrowed) and the two Bacterial Artificial Chromosome clone duplications (BACs RP11-117D22 and RP11-243A18) within 1p32.3 (asterisked). The log2 ratios of the patient vs. reference DNA is shown on the vertical axis (mean log2 ratio of -0.6 for the deletion and 0.38 for the amplification) and the position of each Bacterial Artificial Chromosome along chromosome 1 is shown along the horizontal line. The location of the observed abnormalities is viewed in relation to a chromosome 1 ideogram, with the deletion represented in red and the duplication represented in green.

**Figure 2:**
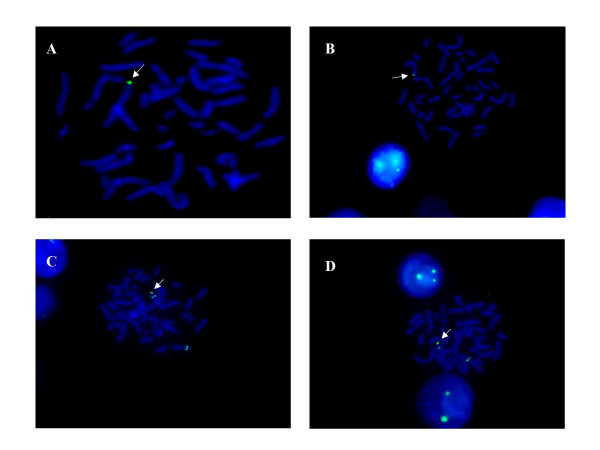
**Metaphase fluorescence in situ hybridisation images**. Metaphase fluorescence in situ hybridisation analysis using the probes for the Bacterial Artificial Chromosome clones RP11-333E3 (A) and RP11-46F15 (B). Both probes (arrowed) show only a single green signal, confirming a deletion within 1p36 (observed in a total of 10 cells). Fluorescence in situ hybridisation analysis using the probes for the Bacterial Artificial Chromosome clones RP11-117D22 (C) and RP11-243A18 (D) both show three green signals (duplicated regions are arrowed), confirming a duplication of the clones within 1p32.3 (observed in a total of 10 cells) and identifying the distal location of the duplicated region within the p-arm of chromosome 1.

The second imbalance was an amplification of two contiguous clones, at 1p32.3 between 0.4 and 1.7 Mb in length. These clones were (in order of most distal): RP11-117D22 and RP11-243A18. The aCGH results for these imbalances of chromosome 1 are illustrated by a scatter plot (Figure 1). The 1p36 deletion was confirmed by FISH using fluorescent probes from two of the five clones (RP11-333E3 and RP11-46F15). Confirmation of the 1p32.3 amplification by FISH (using fluorescent probes from both RP11-117D22 and RP11-243A18) revealed the duplicated segment to be inserted at a position distal to its normal locus, situated within the subtelomeric region of chromosome 1p (Figure 2). Parental samples were requested in order to ascertain the origin of this rearrangement. A paternal sample was unavailable but FISH analysis of the mother, grandmother and siblings of the proband revealed a normal diploid compliment in each case for all of the investigated clones. At present, it cannot be determined whether the chromosomal anomalies found in the proband were the result of a de-novo rearrangement of 1p or familial inheritance from her father.

*In silico *analysis of the deleted and duplicated BAC clones through interrogation of the Ensembl database http://www.ensembl.org/Homo_sapiens/index.html revealed numerous characterised and unknown genes using both the Ensembl and EST transcript gene tracks. Additional file 1 summarises the genes associated with the five deleted BAC clones on 1p36.32 and the two duplicated clones on 1p32.3, in relation to their putative function. Also listed are those genes that lie within the flanking sequences between the deleted clones and neighbouring clones, which may also have been included in the copy number changes.

## Discussion

Monosomy 1p36 is known to cause a range of clinical features including moderate to severe learning difficulties. In this paper, we describe a 9-year-old girl who displayed some of the clinical features associated with deletions of 1p36 and also less well characterised features that may represent a phenotypic manifestation of the 1p32 duplication were identified. Despite similar features identified in other family members, it is thought likely that the deletion and amplification of the specific genes found at the loci described in this patient have contributed to her pronounced clinical phenotype. Gajecka *et al. *[14] categorised the clinical features found in subjects with terminal or interstitial 1p36 deletions. Comparisons of this list, drawn in relation to the proband, confirm the presence of the less well documented phenotypes, including ear asymmetry (40% of patients), clinodactyly (60%), feeding difficulties (77%), reflux (56%), hearing problems (77%) and more commonly speech and developmental delay (100%, 98%) [14]. The presence of the 1p32 amplification in this patient, however, prevents a clear genotype/phenotype association for this subject.

Some of the clinical features listed in the proband are likely to be a result of haploinsufficiency of one or more of the genes identified by *in silico *interrogation of the deleted BAC clones. An example would be to speculate an association between the learning difficulties, developmental delay and speech delay identified in the proband with the deletion of PLCH2. The PLCH2 gene is expressed in mammalian brain tissue including the cerebral cortex [15], a region known to be responsible for memory, thinking and understanding language. Despite the theoretical nature of this approach, this search method can initiate further investigations into such candidate genes.

There are already candidate genes associated with monosomy 1p36 that have been previously correlated with specific phenotypic features (reviewed in [14]). The SKI proto-oncogene is a likely candidate for cleft lip/palate found in 17% of monosomy 1p36 patients. An epilepsy candidate gene KCNAB2 was found to be deleted in the majority of seizure prone 1p36 patients, and human gamma-aminobutyric acid A receptor delta-subunit gene (GABRD) has been implicated in abnormal neurodevelopment. These candidate genes are located at a position distal to the deleted region described in our patient, and may explain the absence of these particular features in our proband.

There is little published material specifically relating to 1p32 duplications. All previous reports encompass much larger duplicated regions of 1p from which a more severe phenotype would be expected in comparison to this case. Therefore, the relevance of this finding with respect to the genes identified within this region is not yet clear.

## Conclusion

It is apparent that with the advent of aCGH as a diagnostic tool for detecting unbalanced genomic rearrangements, it will become easier to correlate specific phenotypic features with the underlying genotype and to identify candidate genes which may be responsible for specific clinical features. This case report provides an example of how identification of small deletions using aCGH may help to dissect out the different phenotypic features of relatively common microdeletion syndromes and facilitate correlation of these with specific genes within the chromosomal regions concerned.

## Consent

Written informed consent was obtained from the patient's family for publication of this case report and any accompanying images. A copy of the written consent is available for review by the Editor-in-Chief of this journal.

## Competing interests

The authors declare that they have no competing interests.

## Authors' contributions

GJF conducted the array CGH work, performed the data analysis relevant to this case report and drafted this manuscript. JCS and SB provided all clinical details and genetic counselling for the patients. SJH performed the checking procedures for this case and revised the manuscript. MMN and GJF conducted the *in silico *data analysis. JKD and GDP carried out the FISH analysis for all patients. JTM performed the required DNA extractions and reviewed the manuscript. LG undertook the aCGH analysis and revised the manuscript.

## Supplementary Material

Additional file 1 Deleted and duplicated genes in the proband. Click here for file
