# Supplementary material for: Array comparative genomic hybridisation-based identification of two imbalances of chromosome 1p in a 9-year-old girl with a monosomy 1p36 related phenotype and a family history of learning difficulties: a case report
Source: J Med Case Rep. 2008 Nov 19;2:355. doi: 10.1186/1752-1947-2-355 (PMC2596801; doi:10.1186/1752-1947-2-355)
Supplement: Additional file 1 — Deleted and duplicated genes in the proband. [file 1752-1947-2-355-S1.doc]

**Table 1.** Deleted and duplicated genes in the proband

|  | **Gene symbol** | **Description/gene name** | **Function** |
| --- | --- | --- | --- |
| **1p36 deleted BAC clones** | | | |
|  | **RER1**† | Retention in endoplasmic reticulum 1 homologue | Involvement in the retrieval of endoplasmic reticulum membrane proteins from the early Golgi compartment |
| **RP3-395M20** | PLCH2 | 1-phosphatidylinositol-4,5-bisphosphate phosphodiesterase eta 2 | PLC(eta)2 is a neuron-specific isoenzyme implicated in development and maintenance of the neuronal network in the postnatal brain |
|  | MMEL1 | Membrane metallo-endopeptidase | Metalloprotease involved in sperm function, possible modulation of fertilisation and early embryonic development. It degrades a wide range of small peptides with an affinity for peptides shorter than 3kDa containing aliphatic or aromatic amino acid residues. Shares the same substrate specificity with MME and cleaves peptides at the same amide bond |
| **RP11-333E3** | ACTRT2 | Actin-related protein T2 | Nucleation of actin filaments including structural roles in mammalian spermatozoa |
|  | PRDM16 | Transcription factor MEL1 | Associated with oncogenesis. MEL1 juxtaposition and overexpression by translocation has been found in cases of myelodysplastic syndromes (MDS), acute non-lymphoblastic leukaemias (ANLL), therapy-related leukaemias and myeloproliferative syndromes |
| **RP4-785P20** | MEGF6 | Multiple epidermal growth factor-like domains 6 precursor (Multiple EGF-like domain protein 3) | EGF-like domains are believed to play a critical role in a number of extracellular events, including cell adhesion and receptor-ligand interactions |
| **RP11-46F15** | WDR8 | WD repeat protein 8. Tryptophan-aspartate repeat containing protein | Proteins belonging to the WD repeat family are involved in a variety of cellular processes, including cell cycle progression, signal transduction, apoptosis, and gene regulation |
|  | TPRG1L/FAM79A | Tumour protein p63 regulated 1-like | A protein known to colocalise with synaptophysin (SYP) located primarily within synaptic vesicles. TPRG1L is thought to function as an effector for CAZ-proteins in vertebrate synapses |
| **RP1-286D6** | TP73 | Tumour protein p73 (p53 like transcription factor) | P73 shares functionality with p53 by induction of apoptosis for multiple cell types |
|  | **CCDC27**† | **Coiled-coil domain-containing protein 27** | Uncharacterised |
|  | **LRRC47**† | **Leucine-rich repeat-containing protein 47** | Uncharacterised |
| **1p32 duplicated BAC clones** | | | |
|  | **SLC1A7**† | **Excitatory amino acid transporter 5** | Involvement in transport of L-glutamate |
| **RP11-117D22** | CPT2 | Carnithine O-palmitoyltransferase 2, mitochondrial precursor | CPT2 protein is located in the inner mitochondrial membrane and is part of the mechanism where long-chain fatty acids undergo beta-oxidation |
|  | C1orf123* | Uncharacterised | Uncharacterised |
|  | MAGOH | mago-nashi homologue, proliferation-associated | Forms part of the conserved protein complex that is involved in mRNA splicing and in the nonsense-mediated decay pathway |
|  | LRP8 | Low density lipoprotein receptor-related protein 8, apolipoprotein e receptor | Functions as a cell surface receptor for both Reelin (RELN) and apolipoprotein E (apoE)-containing ligands |
|  | DMRTB1 | DMRT-like family B with proline-rich C-terminal, 1 | Uncharacterised |
| **RP11-243A18** | GLIS1 | GLIS family zinc finger 1 | GLIS1 protein has a role in controlling gene expression, acting as both a repressor and activator of transcription. Binds to the consensus sequence 5'-GACCACCCAC-3' |
|  | **TMEM48**† | **Nucleoporin NDC1** | A component of the nuclear pore complex (NPC). Involved in NPC and nuclear envelope assembly, possibly by formation of links between the nuclear envelope membrane and soluble nucleoporins, thereby anchoring the NPC in the membrane |
| † | **YIPF1**† | **Protein YIPF1** | Uncharacterised |
|  | **DIO1**† | **Type I iodothyronine deiodinase** | Involved in the deiodination of 3,5,3',5'-tetraiodothyronine (T4) into 3,5,3'-triiodothyronine (T3) and of T3 into 3,3'-diiodothyronine (T2). Plays a role in providing a source of plasma T3 by deiodination of T4 in peripheral tissues such as liver and kidney |
|  | **C1orf41**† | **Placental protein 25** | Uncharacterised |
|  | **LRRC42**† | **Leucine-rich repeat-containing protein 42** | Uncharacterised |
|  | **LDLRAD1**† | **Low-density lipoprotein receptor class A domain-containing protein 1** | Uncharacterised |
|  | **TMEM59**† | **Transmembrane protein 59** | Uncharacterised |
|  | **C1orf83**† | **Uncharacterised protein C1orf83** | Uncharacterised |
|  | **CDCP2**† | **CUB domain-containing protein 2** | Uncharacterised |
|  | **MRPL37**† | **39S ribosomal protein L37** | Uncharacterised |
|  | **C1orf191**† | **Putative uncharacterised protein C1orf191** | Uncharacterised |
|  | **SSBP3**† | **Single-stranded DNA-binding protein 3** | Potentially involved in transcription regulation of the alpha 2(I) collagen gene through binding to the single-stranded polypyrimidine sequences in the promoter region |

A list of the BAC clones and genes found within both the deleted region of chromosome 1p36 and the duplicated region on 1p32 in the proband, relating to the functionality for each

Those genes marked with * do not have Human Genome Organisation (HUGO) assigned gene names. Those genes marked with † are potentially involved in the deleted/duplicated regions as they lie within the flanking sequence between the abnormal clones and the normal neighbouring clones. All information was derived from Online Mendelian Inheritance In Man (www.ncbi.nlm.nih.gov/omim)and Uniprot (www.uniprot.org) both of which provide the reference material for these data.
